# Delay/Disruption Tolerant Networking Performance Characterization in Cislunar Relay Communication Architecture

**DOI:** 10.3390/s25010195

**Published:** 2025-01-01

**Authors:** Ding Wang, Ethan Wang, Ruhai Wang

**Affiliations:** 1 School of Mathematics and Computer Science, Northwest Minzu University, Lanzhou 730030, China; 85142481@xbmu.edu.cn; 2 West Brook High School, Beaumont, TX 77706, USA; wangethan212@gmail.com; 3 Phillip M. Drayer Department of Electrical and Computer Engineering, Lamar University, Beaumont, TX 77710, USA

**Keywords:** space vehicles, space vehicular networks, vehicle-to-everything (V2X) networks, space networks, sensor networks

## Abstract

Future 7G/8G networks are expected to integrate both terrestrial Internet and space-based networks. Space networks, including inter-planetary Internet such as cislunar and deep-space networks, will become an integral part of future 7G/8G networks. Vehicle-to-everything (V2X) communication networks will also be a significant component of 7G/8G networks. Therefore, space networks will eventually integrate with V2X communication networks, with both space vehicles (or spacecrafts) and terrestrial vehicles involved. DTN is the only candidate networking technology for future heterogeneous space communication networks. In this work, we study possible concatenations of different DTN convergence layer protocol adapters (CLAs) over a cislunar relay communication architecture. We present a performance characterization of the concatenations of different CLAs and the associated data transport protocols in an experimental manner. The performance of different concatenations is compared over a typical primary and secondary cislunar relay architecture. The intent is to find out which network relay path and DTN protocol configuration has the best performance over the end-to-end cislunar path.

## 1. Introduction

The National Aeronautics and Space Administration (NASA) is currently in an experimental phase of implementing space communications using computer networking protocols [1]. Transmission Control Protocol (TCP) [2] has severe performance degradation in a space-communications scenario. This is due mainly to significant operating differences between the terrestrial environment and space environment [3,4,5]. Many alternative protocols have been developed for reliable data transport in a space environment [5,6,7,8]; for a comparative discussion of them, refer to the tutorial presented in [9].

Delay/disruption tolerant networking (DTN) [10,11] was developed to combat the long propagation latency and link outages that are typical for the space environment. It is the only candidate networking technology for future heterogeneous space communication networks. Future 7G/8G networks are expected to integrate both terrestrial Internet and space-based networks, including inter-planetary Internet such as cislunar and deep-space networks. In other words, space networks will become an integral part of future 7G/8G networks. The internet of things (IoTs), internet of vehicles (IoVs), vehicle-to-everything (V2X) networks and deep reinforcement learning systems will be of paramount significance to future 7G/8G networks [12,13,14,15]. Furthermore, space networks will eventually integrate with IoTs, IoVs, and V2X communication networks, with both space vehicles (or spacecrafts) and terrestrial vehicles being incorporated.

DTN operates based on a “store and forward” mechanism with an optional custody transfer service [16,17]. DTN’s Bundle Protocol (BP) [18] operates to build a “store and forward” overlay network so that DTN separately implements the “store and forward” mechanism and the optional custody transfer service. The “store and forward” mechanism allows DTN to suspend and resume data transfers when links are interrupted, which are common over space data links. BP has the capability to cope with random and frequent link outages and take advantage of opportunistic connectivity. BP works by utilizing the services of a selected underlying “convergence layer adapter” (CLA) [18,19] and the protocol stack. DTN is designed to operate in this manner because different protocols may be needed for the best performance over different link segments of the end-to-end DTN path. Several CLAs have been developed for BP, and the following three are widely considered for space missions: TCP CLA (i.e., TCPCL) [19], User Datagram Protocol (UDP) CLA (i.e., UDPCL) [20], and Licklider Transmission Protocol (LTP) [21,22,23] CLA (i.e., LTPCL). For a general overview of DTN operation infrastructure and CLAs, refer to [16].

**Figure 1:**
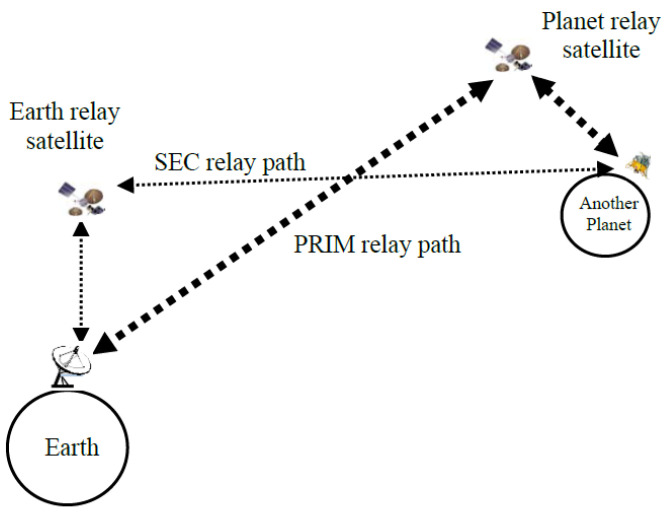
A typical IPN communications relay infrastructure.

Interplanetary communication generally uses relaying to compensate for the limited direct connectivity [24,25]. Almost all the data from the Mars Exploration Rovers (MERs) were sent to Earth via the relaying service of the Mars Global Surveyor and Mars Odyssey orbiter [26]. With the relaying of Mars Odyssey orbiter, the MERs increased their data return from around 30 Mbytes/sol achievable with the direct-to-Earth (DTE) link to around 100~200 Mbytes/sol [27]. The relaying also decreased the power consumption required for data return from roughly 5 Watt hours per Mbyte with the DTE links to around 0.1 Watt hour per Mbyte. For flight missions to the Moon and other planets, similar benefits are also expected. In a typical cislunar and other interplanetary Internet (IPN) [28] communication scenarios, relay-based communication can be operated using two different routing configurations. One configuration is through a relay spacecraft orbiting the planet, and another is through an Earth-orbiting spacecraft, as illustrated in Figure 1. The tracking and data relay satellite system (TDRSS) [29,30] is considered a possible candidate for the relay of cislunar communication, according to [24]. In this work, the path for relaying through the planet (i.e., the Moon) orbiter is considered the primary (PRIM) IPN relay path, while the path for relaying through the Earth-orbiting spacecraft is considered the secondary (SEC) IPN relay path. In Figure 1, cislunar relay communication is carried out only through a single spacecraft. However, multiple spacecrafts could be involved in IPN relaying architectures.

While originally targeting the space environment, extensive work has been carried out regarding the application of DTN in deep-space communications and wireless sensor-based networks. Several countries (notably USA, China, Japan, and India) are working on a Moon landing mission. In this work, we study possible concatenations of different DTN CLAs over a cislunar relay communication architecture. We present a simulation-based performance characterization of the concatenations of different CLAs and the associated data transport protocols in an experimental manner. We intend to find out which network relay path and DTN protocol configuration (or concatenation of them) has the highest goodput performance for data transmission over the end-to-end cislunar path.

The cislunar environment is quite different from deep-space, terrestrial wireless and sensor-based networking environments. This is the first study to incorporate concatenations of different CLAs in cislunar DTN communications and to compare their performance over a typical primary and secondary cislunar relay architecture. The novelty is that this study quantifies the performance of the concatenations of different DTN data transport protocols over different cislunar relay paths using an experimental testbed, with solid statistical data analysis. As the main contribution of the study, this work provides a basis for further investigation and the eventual engineering of DTN-based cislunar communication, which will eventually integrate with future 7G/8G networks, including IoTs, IoVs and V2X communication networks. The investigation results, conclusions and analysis presented in this paper are useful for lunar mission designers when they select networking protocols and cislunar communication relay architectures. They are also expected to be helpful in enhancing DTN protocols for implementing space-based networks.

The related work will be discussed in Section 2. An overview of DTN and its CLAs will be provided in Section 3. In Section 4, we will define the research problem and create hypotheses. In Section 5, the experimental testbed setup and analysis methodology for the proposed study will be discussed. We will present the experimental results in Section 6 and discuss the performance comparison results and the reasons behind the results in Section 7. Then, we will draw the conclusions in Section 8.

## 2. Related Work on Space DTN

DTN was originally developed for space communications. Considering the similarity in communication characteristics, DTN has been extended to terrestrial wireless networks and wireless sensor networks (WSNs) [31].

DTN was introduced to the geo-stationary orbit (GEO)–satellite communication systems in [32]. Some experiment-type work has been conducted for DTN architecture/protocols in satellite and space communications (including deep space). A series of Deep Impact Network Experiments (DINETs) of DTN [33] was conducted by Jet Propulsion Laboratory (JPL). These experiments proved the feasibility of DTN application in deep-space communications, although without a solid performance evaluation. Tests and experimental deployment of DTN were also performed on the International Space Station (ISS) [34,35].

Some analytical studies have also been conducted with DTN-type space network protocols, such as the CCSDS File Delivery Protocol (CFDP), whose functionalities were split between BP and LTP [36,37,38,39]. The reader can refer to [38,39] for the details.

In the past decade, some significant collaborative work has been carried out in developing space networks, including deep-space networks. A part of this work focused on DTN’s LTP [40,41,42,43,44,45], while the rest examined the design and performance of BP [46,47,48,49]. A novel approach for the estimation of bundle delivery time was proposed in [50]. In [51], a model was proposed for a retransmission timer length for BP to optimize the transmission performance. Some studies have explored the impact of link disruption on BP [52,53,54]. Some work has also focused on the energy consumption and transmission overhead of BP [55,56].

However, none of the aforementioned relative work studied the performance of DTN protocols over different cislunar relay paths in a controlled comparative manner.

## 3. Overview of DTN and Its Data Transport Protocols for Space

As mentioned, a store-and-forward transmission mechanism with optional custody transfer is adopted by DTN to deal with frequent link outages and long data delivery latency [18]. Similar to the operation of traditional postal systems, the mechanism moves each data fragment hop by hop until the entirety of the source data are delivered to the destination [16]. To implement the store-and-forward transmission service, BP [18] operates on top of underlying heterogeneous CLP stacks (together with various data transport and Internet protocols) to build an overlay network.

**Figure 2:**
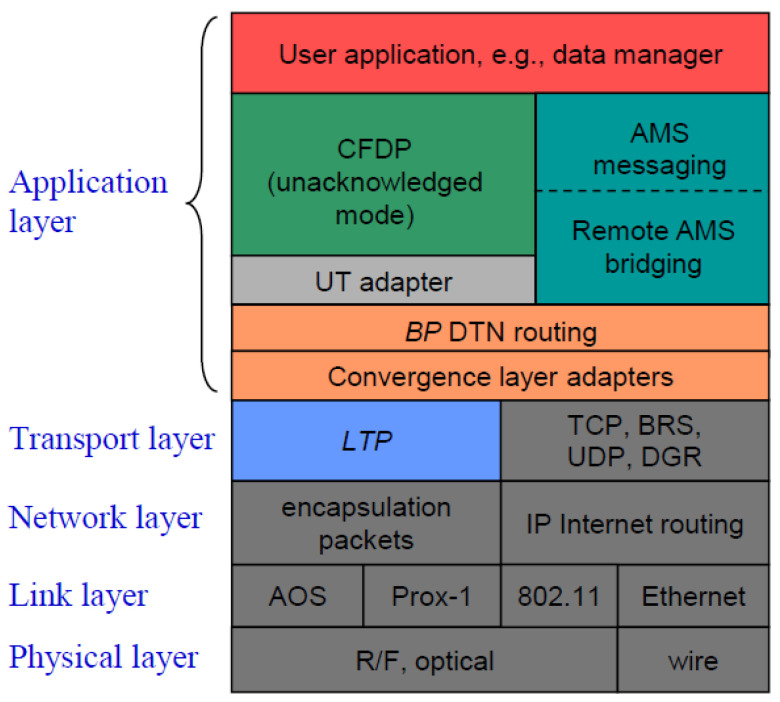
DTN protocol stack vs. OSI stack [57].

The DTN protocol stack (including the specific protocols) compared to the seven-layer OSI model [57] is illustrated in Figure 2. As observed, the DTN protocol architecture is similar to the one of the Internet. As IP interconnects physical networks such as the Ethernet or SONET, BP interconnects “internets”, including both Internet-based terrestrial networks (e.g., IEEE 802.11 and Ethernet) and space-based networks built over space links such as the Advanced Orbiting Systems (AOS) and Proximity-1.

As introduced, convergence-layer adapters for a variety of transport protocols and implementations have been developed to support DTN operation in heterogeneous networks, including TCPCL, UDPCL and LTPCL. As shown in Figure 2, these CLAs and their associated data transport protocols locate between BP and the network/data link layers.

For TCPCL and UDPCL of DTN, as the names imply, they are designed to operate over TCP or UDP, respectively. In other words, they use TCP or UDP to provide reliable or unreliable communication services between DTN nodes [19].

Compared to TCP and UDP, LTP for LTPCL was developed as the primary transport protocol of space DTN. LTP was designed to provide selectable transmission service according to mission requirements. In other words, LTP can provide reliable transmission service (as TCP does) and unreliable service (as UDP does) [22]. Both transmission services can be carried out for a single data block by specifying the “red” portion (for reliable service) of data or the “green” portion (for unreliable service) of data. Unlike the basic UDPCL, LTP supports the segmentation of large DTN bundles for transmission in multiple UDP datagrams. To accommodate the highly asymmetric data rates of the down link and forward link, some protocol designers try to reduce the amount of ACK traffic over the constrained ACK channel. For LTP, this is realized by aggregating multiple BP bundles into a single LTP block that is acknowledged at block granularity [57]. The protocol design and operation of LTP are well discussed in previous studies [22]; therefore, they are not discussed in detail in this paper.

TCP and UDP show high performance for data/file transfer over terrestrial links—as they have consistent link connectivity and long latency—but not over space links. In comparison, it is expected that LTP works effectively over a space link characterized by intermittent connectivity, long link outage and long propagation delay. Therefore, it is reasonable to concatenate two or three protocols for optimal data/file transfer in cislunar and deep-space communications. For example, for the end-to-end transfer from the Moon to an Earth ground station, TCPCL or UDPCL should likely be considered for use over the link between the data node on the surface of Moon to its orbiter, which has negligible delay. In contrast, as the link between the Moon orbiter and the data destination node on the Earth ground station has a long propagation delay and intermittent connectivity, LTPCL should be adopted.

In next section, we discuss each of the DTN CLA configurations, including their concatenations and their operational architecture, in cislunar communications that we adopted for the investigation in this work.

## 4. Research Problem and Hypotheses Definition

This work is devoted to the performance characterization of various DTN CLA selections in a controlled simulation manner in typical primary and secondary cislunar relay communications architectures. We intend to find an answer to the following problem: Which configuration of DTN CLAs and cislunar relay path has the highest goodput in cislunar communications? To find an answer, we select protocol comparison pairs based on discussions with DTN protocol developers and run simulations for file transfer over both primary and secondary cislunar relay architectures. We then compare their performance.

**Figure 3:**
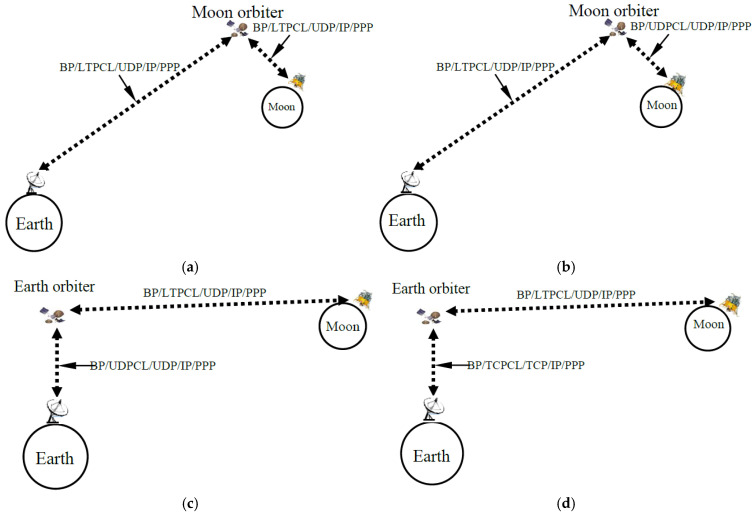
Comparison of simulated cislunar communication relay architecture and DTN protocol configurations. (**a**) LTP LTP PRIM. (**b**) UDP LTP PRIM. (**c**) LTP UDP SEC. (**d**) LTP TCP SEC.

In comparison to TCP and UDP, which are mainly designed to operate over short-delay and less lossy links, the newly developed LTP targets long-haul links. Therefore, for all the protocol configurations in this work, BP/LTPCL is configured to run over UDP/IP/PPP (i.e., BP/LTPCL/UDP/IP/PPP) for file transfer over the long-delay cislunar links either from the Moon orbiter to the Earth ground station or from the Moon surface to the Earth orbiting satellite. In contrast, either BP/TCPCL/TCP/IP/PPP or BP/UDPCL/UDP/IP/PPP is adopted for the proximity links from the Moon surface to the Moon orbiter or from the Earth orbiter to the Earth ground station. Specifically, as illustrated in Figure 3, the file transfer simulations are run with four hybrids of DTN protocol configurations over two cislunar relay architectures (i.e., the primary (PRIM) and secondary (SEC) relay paths shown in Figure 1). Each of four configurations in Figure 3 is clarified below:

- LTP LTP PRIM (or simply L_L_P) in Figure 3a—For this configuration, we conduct a DTN test over the PRIM relay path in Figure 1, with BP/LTPCL/UDP/IP/PPP running over both link segments from TX to MX (i.e., from Moon to its orbiter) and from MX to RX (i.e., from a Moon orbiter to Earth ground station), i.e., a single protocol stack, BP/LTPCL/UDP/IP/PPP, runs over both links of a typical primary relay path.
- UDP LTP PRIM (or simply U_L_P) in Figure 3b—For this configuration, we conduct a DTN test over the PRIM relay path in Figure 1, with BP/UDPCL/UDP/IP/PPP running from TX to MX (i.e., from Moon to its orbiter) and BP/LTPCL/UDP/IP/PPP running from MX to RX (i.e., from a Moon orbiter to Earth ground station).
- LTP UDP SEC (or simply L_U_S) in Figure 3c—For this configuration, we conduct a DTN test over the SEC relay path in Figure 1, with BP/LTPCL/UDP/IP/PPP running from TX to MX (i.e., from Moon to an Earth orbiter) and BP/UDPCL/UDP/IP/PPP running from MX to RX (i.e., from an Earth orbiter to Earth ground station).
- LTP TCP SEC (or simply L_T_S) in Figure 3d—For this configuration, we conduct a DTN test over the SEC relay path in Figure 1, with BP/LTPCL/UDP/IP/PPP running from TX to MX (i.e., from Moon to an Earth orbiter) and BP/TCPCL/TCP/IP/PPP running from MX to RX (i.e., from an Earth orbiter to Earth ground station).

A null hypotheses, *H*_0_, and an alternative hypotheses, *H*_1_, are created for each comparison pair for each channel condition of the BERs and link delays. The null hypothesis is that two configurations, when compared, do not have a significant goodput performance difference, while the alternative null hypothesis is that two configurations, when compared, have a significant goodput performance difference.

Based on the protocol choices and communication relay architectures, the following comparison pairs and corresponding null hypotheses and alternative hypotheses are created:

(1) Comparison between LTP LTP PRIM and UDP LTP PRIM.
  $$H_{0}:\mu_{L\_L\_P}=\mu_{U\_L\_P}\;\mathrm{versus}\;H_{1}:\mu_{L\_L\_P}\neq \mu_{U\_L\_P}$$
(2) Comparison between LTP LTP PRIM and LTP TCP SEC.
  $$H_{0}:\mu_{L\_L\_P}=\mu_{L\_T\_S}\;\mathrm{versus}\;H_{1}:\mu_{L\_L\_P}\neq \mu_{L\_T\_S}$$
(3) Comparison between LTP TCP SEC and LTP UDP SEC.
  $$H_{0}:\mu_{L\_T\_S}=\mu_{L\_U\_S}\;\mathrm{versus}\;H_{1}:\mu_{L\_T\_S}\neq \mu_{L\_U\_S}$$
(4) Comparison between LTP TCP SEC and UDP LTP PRIM.
  $$H_{0}:\mu_{L\_T\_S}=\mu_{U\_L\_P}\;\mathrm{versus}\;H_{1}:\mu_{L\_T\_S}\neq \mu_{U\_L\_P}$$
(5) Comparison between LTP UDP SEC and UDP LTP PRIM.
  $$H_{0}:\mu_{L\_U\_S}=\mu_{U\_L\_P}\;\mathrm{versus}\;H_{1}:\mu_{L\_U\_S}\neq \mu_{U\_L\_P}$$

## 5. Testbed Setup and Analysis

### 5.1. Testbed Setup

**Figure 4:**
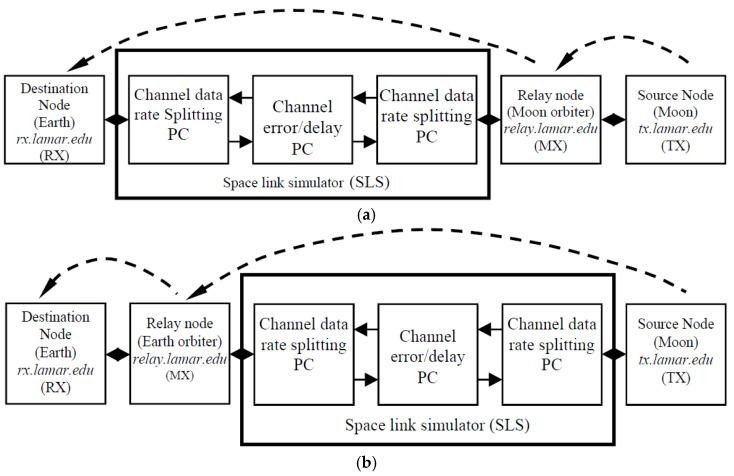
SCNT block diagram. (**a**) For primary cislunar relay architecture. (**b**) For secondary cislunar relay architecture.

In Figure 4, we show a block diagram of the Space Communication and Networking Testbed (SCNT) adopted for the experimental file transfer in this study. The SCNT was designed to emulate the relay type of IPN communication scenarios via the primary relay path and the secondary relay path illustrated in Figure 1. As presented in Figure 4, three Linux-based PCs—TX, MX and RX—simulate the data source on the planeting ude ated in Figure 1. Via the primary relay ath, a planet orbiter (in Figure 4a) or an Earth orbiter (in Figure 4b), and a ground station on the Earth, respectively. The testbed was validated in our previous research [8,37,40]. For the details of SGLS operation and better explanation of the simulation parameters, refer to [37,40].

The major experimental configurations are listed in Table 1. The experimental factors and their settings are straightforward and clear. As a matter of fact, the one-way light time in cislunar communication is at least 1.25 s, while it can be 5 s at maximum [24]. Following this fact, we choose nine different cislunar long-haul delays (i.e., the delays for MX → RX in Figure 4a and TX → MX in Figure 4b), as listed in Table 1, for the performance evaluation of DTN. Considering the proximity link delays (i.e., the delays for Moon → Moon orbiter and Earth orbiter → Earth) are so short that they can be negligible compared to the long-haul delays, they are ignored in the simulation setup.

The PPP link rate of 115,200 bit/s is chosen for both data and ACK channels. This rate is common for cislunar communications [58,59]. Table 2 shows the data link rates and propagation delay experienced in a few actual lunar exploration missions. The experimental parameters in this work fall within the range of the cislunar communication parameters of actual missions in Table 2, and therefore, their selections are practical. The maximum and default sender/receiver buffer size of 16 Mbytes is configured for TX, MX and RX. This buffer size is big enough to accommodate the data transmission, with a large BDP caused mainly by the mentioned long link delays.

### 5.2. Analysis Methodology

According to our simulation results, the goodput performance of each protocol stack is observed from approximately normal populations. With this observation, the statistical *t*-statistic [60] was adopted for pair-wise comparisons among the protocol stacks.

As mentioned, we are interested in a performance comparison of four DTN protocol configurations—LTP LTP PRIM (L_L_P), UDP LTP PRIM (U_L_P), LTP UDP SEC (L_U_S) and LTP TCP SEC (L_T_S)—for each combination of BER and link delay.

Let $W_{L\_L\_P\_1},W_{L\_L\_P\_2},\ldots ,W_{L\_L\_P\_16}$ be sixteen (16) samples of goodput performance from LTP LTP PRIM normal populations with mean $\mu_{L\_L\_P}$ for each combination of three BERs and nine link delays.

Let $X_{U\_L\_P\_1},X_{U\_L\_P\_2},\ldots ,X_{U\_L\_P\_16}$ be sixteen (16) samples of goodput performance from UDP LTP PRIM normal populations with mean $\mu_{U\_L\_P}$ for each combination of three BERs and nine link delays.

Let $Y_{L\_U\_S\_1},Y_{L\_U\_S\_2},\ldots ,Y_{L\_U\_S\_16}$ be sixteen (16) samples of goodput performance from LTP UDP SEC normal populations with mean $\mu_{L\_U\_S}$ for each combination of three BERs and nine link delays.

Let $Z_{L\_T\_S\_1},Z_{L\_T\_S\_2},\ldots ,Z_{L\_T\_S\_16}$ be sixteen (16) samples of goodput performance from LTP TCP SEC normal populations with mean $\mu_{L\_T\_S}$ for each combination of three BERs and nine link delays.

Then, the *t*-test statistic for each pair can be formulated as

$$t_{L\_L\_P-U\_L\_P}=\frac{{\bar{W}}_{L\_L\_P}-{\bar{X}}_{U\_L\_P}}{\sqrt{s_{L\_L\_P}^{2}/n_{L\_L\_P}+s_{U\_L\_P}^{2}/n_{U\_L\_P}}}$$

$$t_{L\_L\_P-L\_T\_S}=\frac{{\bar{W}}_{L\_L\_P}-{\bar{Z}}_{L\_T\_S}}{\sqrt{s_{L\_L\_P}^{2}/n_{L\_L\_P}+s_{L\_T\_S}^{2}/n_{L\_T\_S}}}$$

$$t_{L\_T\_S-L\_U\_S}=\frac{{\bar{Z}}_{L\_T\_S}-{\bar{Y}}_{L\_U\_S}}{\sqrt{s_{L\_T\_S}^{2}/n_{L\_T\_S}+s_{L\_U\_S}^{2}/n_{L\_U\_S}}}$$

$$t_{L\_T\_S-U\_L\_P}=\frac{{\bar{Z}}_{L\_T\_S}-{\bar{X}}_{U\_L\_P}}{\sqrt{s_{L\_T\_S}^{2}/n_{L\_T\_S}+s_{U\_L\_P}^{2}/n_{U\_L\_P}}}$$

$$t_{L\_U\_S-U\_L\_P}=\frac{{\bar{Y}}_{L\_U\_S}-{\bar{X}}_{U\_L\_P}}{\sqrt{s_{L\_U\_S}^{2}/n_{L\_U\_S}+s_{U\_L\_P}^{2}/n_{U\_L\_P}}}$$

where

${\bar{W}}_{L\_L\_P}$ denotes the goodput sample mean for LTP LTP PRIM;

${\bar{X}}_{U\_L\_P}$ denotes the goodput sample mean for UDP LTP PRIM;

${\bar{Y}}_{L\_U\_S}$ denotes the goodput sample mean for LTP UDP SEC;

${\bar{Z}}_{L\_T\_S}$ denotes the goodput sample mean for LTP TCP SEC;

S… denotes the standard deviation of the goodput for each protocol configuration;

n… denotes the sample size for each protocol configuration. This size is chosen as sixteen (16) in this work according to Fisher’s comparison procedure [60] based on $\left|\bar{y_{i}}-\bar{y_{j}}\right|\geq t_{\alpha /2,a(n-1)}\sqrt{\frac{2MS_{e}}{n}}$.

The *p*-value for each comparison pair is computed and found using the Student’s *t* table [60]. The *p*-value serves as a measure of the evidence against the null hypothesis *H*_0_ [61], as clarified below:

- The test result of (*p* < 0.01) provides very strong evidence to reject *H*_0_, implying significant goodput difference between the two configurations.
- The test result of (0.01 ≤ *p* < 0.05) provides moderate evidence to reject *H*_0_, implying reasonable goodput difference between the two configurations.
- The test result of (0.05 ≤ *p* < 0.1) provides suggestive evidence to reject *H*_0_, implying implicative goodput difference between the two protocols.
- The test result of (*p* > 0.1) provides no real evidence to reject *H*_0_, implying no goodput difference between the two protocols.

## 6. Performance Evaluation Results

In this section, the goodput performance results of three CLA protocols and their concatenations over the primary and the secondary cislunar communication relay architecture are presented.

### 6.1. Comparison with a BER of 0

**Figure 5:**
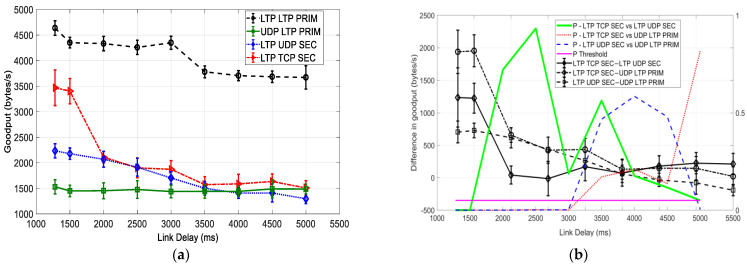
Goodput (vs. link delay) comparison of three different protocols and their hybrid over the primary and secondary cislunar communication paths with a BER of 0. (**a**) Goodput. (**b**) Goodput differences and *p*-values.

Figure 5 illustrates a comparison of the goodput (vs. delay) of three different protocol options and their hybrid for file transmission over the primary and secondary cislunar communication paths with a BER of 0. In Table 3, the notations in Figure 5b are described.

As observed from Figure 5a, the goodput of the LTP LTP PRIM is the highest at all the experimented delay settings. LTP TCP SEC also shows high goodput, but only at delays of 1280 ms and 1500 ms. Along with the increase in delay, the goodput drastically drops and binds LTP UDP SEC and UDP LTP PRIM. In comparison, UDP LTP PRIM has the lowest consistently at all link delays—around 1500 bytes/s.

For a hybrid of two protocols over primary and secondary paths, the goodput of LTP UDP SEC is comparatively higher than UDP LTP PRIM for the link delays up to 3000 ms. For a longer delay, they show about equal performance. This observation is supported by statistical analysis in Figure 5b. *P*-LTP UDP SEC vs. UDP LTP PRIM is smaller than 0.01 for the link delays of 1280 ms~3000 ms. This provides very strong evidence to reject *H*_0_, implying significant goodput difference between LTP UDP SEC and UDP LTP PRIM for this range of delay. We also see that LTP UDP SEC–UDP LTP PRIM remains positive, decreasing from 700 bytes/s to 50 bytes/s, in this range of link delay. This explains why LTP UDP SEC has significant goodput advantage over UDP LTP PRIM for the simulated link delays of 1280 ms~3000 ms. For the delays of 3500 ms~4500 ms, *P*-LTP UDP SEC vs. UDP LTP PRIM is around 0.5, providing no real evidence to reject *H*_0_ and no significant goodput difference between two configurations.

For a comparison between LTP UDP SEC and LTP TCP SEC, we see that LTP TCP SEC has higher goodput for delays around 1500 ms or shorter. As the delay increases, the performance difference between the two protocol transmissions disappears. The *p*-values in Figure 5b prove this. *P*-LTP TCP SEC vs. LTP UDP SEC is smaller than 0.01 for the delays of 1500 ms or shorter. This is very strong evidence to reject *H*_0_, which implies a significant performance difference between LTP TCP SEC and LTP UDP SEC. The numerical value of LTP TCP SEC–LTP UDP SEC is around 1200 bytes/s for this range of delay, which is the goodput advantage of LTP TCP SEC over LTP UDP SEC. The *p*-values are mostly greater than 0.1 for delays around 2000 ms or longer, indicating that the two protocols have no significant goodput difference.

As we also observed, LTP LTP PRIM has a significant advantage over UDP LTP PRIM throughout all delays. For this comparison, we ran LTPCL/UDP for a long link from MX to RX and ran two different protocols for the short delay from TX to MX, i.e., one running LTPCL/UDP and another running UDPCL/UDP. The performance difference implies that UDPCL/UDP running over a short-delay link affects the performance of the LTPCL/UDP running over a long-delay link, resulting in performance degradation for the complete connection.

Please note that LTP LTP PRIM is not involved in the comparison with other protocol options in Figure 5b. This is because it is obvious in Figure 5a that LTP LTP PRIM has significant goodput advantage over any other protocols. There is no need for any verification.

### 6.2. Comparison with a BER of 10^−6^

**Figure 6:**
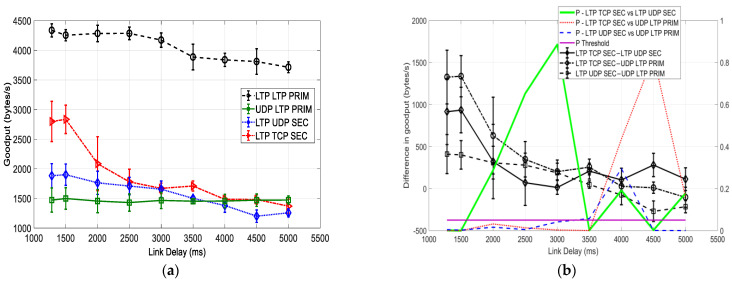
Goodput (vs link delay) comparison of three different protocols and their hybrid over the primary and secondary cislunar communication paths with a BER of 10^−6^. (**a**) Goodput. (**b**) Goodput differences and *p*-values.

Figure 6 presents a comparison of the goodput (vs delay) of three different protocol options and their hybrid for file transmission over the primary and secondary cislunar communication paths with a BER = 10^−6^. A general observation in Figure 6a, in comparison with a BER of 0, is that the goodput of most DTN protocol options slightly decreases at all the link delays because of bundle corruptions caused by the introduction of a channel BER of 10^−6^. An exception happens for the performance of UDP LTP PRIM, which remains about the same as at a BER of 0. This implies that the performance of UDP LTP PRIM is not affected by link delay and channel noise. This is because its file transfer is mainly controlled by unreliable UDP-based transmission. However, in comparison to a BER of 0, the overall performance rank of all four protocol options remains about the same. This is because the increase in BER from 0 to 10^−6^ does not affect the data transmission severely, as only a very limited number of bundles are corrupted on average.

Similar to Figure 5a, a direct observation in Figure 6a is that LTP LTP PRIM outperforms all other protocol options throughput all the link delays over both relay paths, with an even more significant performance advantage than that with a BER = 0. With the increase in the delay, the goodput of other three protocol configurations merges. Figure 6b provides statistical analysis results for this observation. We observe that both *P*-LTP TCP SEC vs. UDP LTP PRIM and *P*-LTP UDP SEC vs. UDP LTP PRIM tend to increase from about 0 to above 0.1 for the delay increase from 1280 ms to around 4500 ms. In addition, the absolute goodput differences between each pair of protocols (i.e., LTP TCP SEC–LTP UDP SEC, LTP TCP SEC–UDP LTP PRIM, and LTP UDP SEC–UDP LTP PRIM) drop from a range of 1300~450 bytes/s to around 0 bytes/s.

As shown in Figure 5b, LTP LTP PRIM is not compared with any other protocol options in Figure 6b because its goodput advantage over others is obvious.

With the increase in the delay, the goodput of LTP TCP SEC drops drastically, but it is still the second highest for the delays of 1280 ms~3500 ms. As discussed, UDP LTP PRIM shows consistent performance at all the link delays, and it has the lowest goodput in the delay range of 1280 ms~3500 ms. *P*-LTP TCP SEC vs. UDP LTP PRIM in Figure 6b indicates that the *p*-value of their difference is originally very low (below 0.05) up to 3500 ms, but it increases to 0.4, 0.8 and 0.16 at 4000~5000 ms, respectively. This explains that LTP TCP SEC has significant goodput advantage over UDP LTP PRIM for short delays up to 3500 ms, but the advantage disappears for longer link delays. A similar result is observed for the comparison between LTP UDP SEC and UDP LTP PRIM, as illustrated by *P*-LTP UDP SEC vs. UDP LTP PRIM. For the link delay longer than 3500 ms, LTP UDP SEC drops below UDP LTP PRIM—we observed that LTP UDP SEC–UDP LTP PRIM changes roughly linearly from the initial 450 bytes/s to −250 bytes/s.

### 6.3. Comparison with a BER of 10^−5^

**Figure 7:**
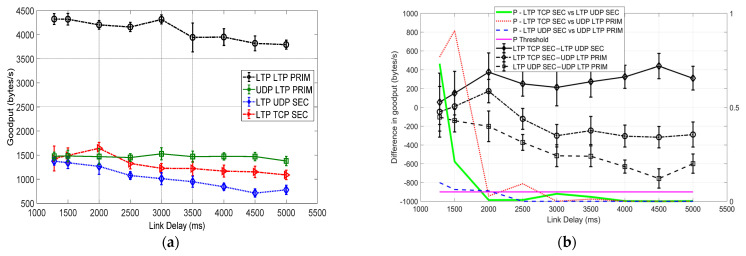
Goodput (vs. link delay) comparison of three different protocols and their hybrid over the primary and secondary cislunar communication paths with a BER of 10^−5^. (**a**) Goodput. (**b**) Goodput differences and *p*-values.

Figure 7 presents a goodput comparison of the DTN different protocols with a BER of 10^−5^. Similar to the transmissions at BERs of 0 and 10^−6^, LTP LTP PRIM shows a significant advantage at all link delays. In comparison, the performance advantage is more significant than that at BERs of 0 and 10^−6^. Also, different from the comparisons at BERs of 0 and 10^−6^, the performance of UDP LTP PRIM, LTP UDP SEC and LTP TCP SEC binds at short link delays (<2000 ms). This is clearly seen from the statistical analysis in Figure 7b in which *P*-LTP TCP SEC vs. LTP UDP SEC, *P*-LTP TCP SEC vs. UDP LTP PRIM, and *P*-LTP UDP SEC vs. UDP LTP PRIM are either greater than 0.5 or greater than 0.05 at most link delays. This indicates that UDP LTP PRIM, LTP UDP SEC and LTP TCP SEC basically have no significant performance difference or have only implicative differences.

However, the performance lines of the investigated protocols tend to separate each other and show difference with the increase in the delay (≥2000 ms), as observed in Figure 7a. This is verified from Figure 7b, in which *P*-LTP TCP SEC vs. UDP LTP PRIM, *P*-LTP TCP SEC vs. UDP LTP PRIM, and *P*-LTP UDP SEC vs. UDP LTP PRIM are mostly less than 0.05 or even less than 0.01. This provides moderate or strong evidence to reject *H*_0_ and shows the reasonable or significant goodput difference between each pair.

Different from the comparison at BERs of 0 and 10^−6^ in which LTP TCP SEC shows a slightly higher goodput than the other two, UDP LTP PRIM has goodput advantage over both LTP UDP SEC and LTP TCP SEC at most delays with a BER = 10^−5^. The advantage basically increases with the delay increase from 2000 ms to 5000 ms. This can be observed from a comparison of the absolute performance difference in Figure 7b, which shows that the goodput difference between LTP TCP SEC–UDP LTP PRIM and LTP UDP SEC–UDP LTP PRIM increases along with the increase in the delay from 2000 ms to 5000 ms. LTP UDP SEC show the poorest performance compared to any other protocols at all link delays. In comparison, LTP UDP SEC has higher goodput than UDP LTP PRIM at most link delays at BERs of 0 and 10^−6^, especially for short delays ≤ 3000 ms.

Similar to BERs of 0 and 10^−6^, UDP LTP PRIM shows consistent goodput performance at around 1500 bytes/s. In comparison, LTP UDP SEC and LTP TCP SEC show obvious performance degradation along with the increase in the delays at all three BERs.

## 7. Discussions and Clarification of Comparison Results

The performance comparison results and CLA configurations are discussed in this section. The experimented protocol configurations are ranked first according to their performance evaluation results. Then, a comprehensive discussion of the CLA operation and performance comparison is provided to clarify why they performed differently.

The investigated DTN protocols and configurations are roughly ranked in Table 4. according to their goodput performance at all three BERs. As observed, LTP LTP PRIM (i.e., running LTP over both link segments of the primary relay path) is ranked on top among all four protocol configurations, followed by LTP TCP SEC, then by the transmissions involving UDP (i.e., UDP LTP PRIM and LTP UDP SEC).

### 7.1. Cislunar (Space) Segment of the End-to-End Path

For all four configurations, the long-delay cislunar segment is involved, and it always has the same configurations:

- Always running LTP over UDP/IP/PPP;
- Always 115,200 bps;
- Varying signal propagation delay (1280 to 5000 ms each way);
- Varying BER (0 to 10^−5^).

As discussed in Section 4, LTPCL/UDP/IP/PPP is always configured for the long-delay cislunar segment (either Moon orbiter → Earth ground station or Moon surface → Earth orbiter) with the maximum and default sender/receiver buffer size of 16 Mbytes for all three stations, so this segment behaves in the same way in each configuration.

End-to-end goodput is reduced as signal propagation delay increases. The root cause of this effect is that the number of block transmission sessions this LTP implementation can conduct concurrently is limited to a fixed maximum *N* established at system initialization (This is to prevent LTP from exceeding available storage and memory resources). This limit means that as soon as *N* sessions have been initiated, no new transmission session can be begun until one of the existing sessions completes. As signal propagation delay increases the length of time required to complete a session (i.e., receive a positive ACK from the receiver) increases, and therefore, the time at which new transmission can be initiated is postponed, overall latency for the exercise increases, and goodput drops.

The mitigation for this effect is to increase *N* so that by the time the *N*th session is being initiated the first session has already completed, so that there is never any delay in initiating a new session. However, this strategy solves the problem only when there is never any data loss: data loss will delay the completion of a session by one or more round-trips, delaying the initiation of new sessions regardless of the session limit, thereby increasing overall latency and reducing goodput.

Therefore,

Whenever the rate of data loss on the cislunar segment is greater than zero, end-to-end goodput drops as signal propagation delay on the cislunar segment increases. Because this data loss is random, the drop in end-to-end goodput due to this effect is not deterministic, but the trend is clear.

This drop in goodput with increasing delay is especially pronounced when commanded data loss, due to changes in the commanded BER, is introduced. However, the effect is noticeable even when the commanded BER is zero, because one additional source of data loss remains: since LTP is running over UDP/IP/PPP, which lacks congestion control, there can always be UDP congestion loss on the cislunar segment. This is data loss that is an artifact of the testbed rather than a controlled variable.

Note, though, that the severity of the end-to-end goodput decline due to data loss on the cislunar segment varies with the rate at which traffic on that segment is initiated, as discussed below.

### 7.2. Primary Configurations (i.e., LTP LTP PRIM and UDP LTP PRIM)

In the two primary configurations, the behavior of the end-to-end path is determined by the nature of the proximity segment from the lunar surface to the lunar orbiter. This is because in both configurations, the rate of initiation of traffic on the cislunar segment is limited by the rate at which data arrive at the orbiter over the proximity segment.

#### 7.2.1. LTP on the Proximity Segment (i.e., LTP LTP PRIM)

In this configuration, there may be congestion loss due to the use of UDP/IP/PPP under LTP on the proximity segment. The rate of data arrival at the orbiter—and hence the rate of initiation of traffic on the cislunar segment—is still high, though: when there is congestion loss on the proximity segment, the signal propagation delay on this segment is so low that LTP retransmission and successful delivery are very rapid.

However, the operation of LTP on the proximity segment has the effect of “screening” much of the potential congestion loss out of the cislunar segment. The effective rate of arrival of data at the orbiter is by definition reduced to a level that introduces no further congestion loss. Since this data arrival rate is the upper limit of the rate at which traffic on the cislunar segment can be initiated, the opportunity for further congestion loss on the cislunar link is minimized.

Since (a) there is no uncorrected data loss on either segment, (b) congestion loss on the proximity segment introduces little goodput degradation due to the low signal propagation delay, and (c) congestion loss on the cislunar segment is minimized, there is little net goodput degradation on the end-to-end path. This configuration may be expected to exhibit the best performance among the four.

#### 7.2.2. UDP on the Proximity Segment (i.e., UDP LTP PRIM)

UDP/IP lacks native rate control or congestion control: whenever the data rate of the underlying link service exceeds the rate at which the receiving UDP/IP implementation can receive and process inbound datagrams, newly received datagrams will overwrite unprocessed reception buffer contents, causing datagram loss due to congestion. The rate of UDP congestion loss in our simulations would have been lower if we were running UDP/IP over a slower link, because datagrams would never arrive at rates in excess of the rate at which the protocol stack could handle them. However, because we were running at relatively high data rates, UDP/IP congestion loss was significant. This high rate of data loss affected not only the nominal UDP/IP (proximate) segments of the end-to-end paths but also the LTP (cislunar space) segments, because the space link protocol underlying LTP was simulated by, again, UDP/IP. In other words, for the configuration of UDP LTP PRIM, congestion loss in the transmission of data from the lunar surface to the orbiter by UDP (without any recovery from data loss, as would be provided by LTP) means that the rate of data arrival at the orbiter is significantly lower than the rate at which the application produces data for transmission. The resulting rate of initiation of traffic on the cislunar segment is in fact so low that congestion loss on the cislunar segment is virtually eliminated: the receiving UDP on the cislunar segment is almost always able to process received datagrams before they can be overwritten by subsequently received datagrams. This accounts for the low but generally flat goodput curves for this configuration: the constant low rate of data arrival (due to uncorrected data loss) at the orbiter dominates, precluding significant congestion loss on the cislunar segment and a resulting decline in goodput.

### 7.3. Secondary Configurations (i.e., LTP UDP SEC and LTP TCP SEC)

In the two secondary configurations, the behavior of the end-to-end path is largely independent of the nature of the proximity segment from the Earth orbiter to the surface of Earth. This is because, in these configurations, the rate of initiation of traffic on the cislunar segment is simply the rate at which the application produces data for transmission, i.e., the maximum possible rate imposed by this exercise. This results in the maximum opportunity for congestion loss on the cislunar segment and therefore the maximum possible goodput decline due to this effect, and this decline is exhibited no matter what protocol is used on the proximity segment.

Note that the effective rate of arrival of data at the orbiter is determined by the rate at which recovery from data loss can be performed on the cislunar segment, which declines with increasing signal propagation delay (as discussed above). The roles of the cislunar segment and proximity segment are the reverse of LTP LTP PRIM, in that the cislunar segment’s congestion loss and recovery “screens” most potential congestion loss out of the proximity segment. Therefore, the opportunity for congestion loss on the proximity link is generally low and drops with increasing signal propagation delay (resulting in decreasing data arrival rate) on the cislunar segment.

#### 7.3.1. UDP on the Proximity Segment (i.e., LTP UDP SEC)

In this configuration (as discussed for UDP LTP PRIM in subsection B-2), significant congestion losses happen in the transmission of data from the Earth orbiter to the surface of Earth by UDP (without any recovery from data loss, as would be provided by LTP). This means that the rate of data arrival at the surface is always lower than the rate at which the cislunar segment delivers data to the orbiter. That accounts for the manner in which the goodput curve for LTP LTP PRIM is roughly the same as the curve for LTP LTP PRIM, but with a negative offset. The effective rate of arrival of data at the orbiter is initially low and decreases with increasing signal propagation latency on the cislunar segment, so the opportunity for congestion loss on the proximity link is minimized, but any congestion loss that does occur is uncorrected and further degrades goodput. This is the worst-case scenario.

#### 7.3.2. TCP on the Proximity Segment (i.e., LTP TCP SEC)

In this configuration, the use of TCP for the transmission of data from the Earth orbiter to the surface of Earth precludes data loss on the proximity segment. Again, the cislunar segment’s congestion loss and recovery “screens” most potential congestion loss out of the proximity segment. Unlike in the configuration of LTP UDP SEC, any congestion loss that does occur on the proximity segment is immediately corrected by TCP, so the performance of LTP TCP SEC will always be somewhat better than that of LTP UDP SEC. However, as congestion loss increases due to increasing signal propagation delay on the cislunar segment, the rate of data arrival at the orbiter drops and the opportunity for congestion loss on the proximity segment decreases, reducing the relative advantage of TCP over UDP on that segment.

## 8. Conclusions

In this paper, a simulation-based performance characterization of the DTN architecture and protocols over the primary and secondary cislunar relay architecture is presented. The focus was on data transmission from spacecrafts to Earth ground stations over the end-to-end cislunar communication path. The investigated DTN protocol configurations are roughly ranked according to their goodput performance. Based on the investigation results, LTP LTP PRIM (i.e., running LTP over both link segments of the primary cislunar relay path) shows a significant performance advantage over any other DTN protocol configurations at all cislunar link delays (1200 ms~5000 ms) and noise levels with a BER of 0~10^−5^. For the transmission over the secondary relay path, LTP TCP SEC (i.e., running LTP over the long link segment and TCP over the short link segment of the secondary relay path) has an overall performance advantage over LTP UDP SEC (i.e., running LTP over the long link segment and UDP over the short link segment of the secondary relay path). However, the performance advantage is significant only over less lossy cislunar channels with a BER ≤ 10^−6^ accompanied by a short link delay (<2000 ms) and over lossy cislunar channels with a BER = 10^−5^ accompanied by a long link delay (≥2000 ms). In all other investigated cislunar communications scenarios, the goodput advantage of LTP TCP SEC over LTP UDP SEC is not considered significant.

UDP LTP PRIM show the lowest goodput amongst all the DTN protocol configurations for link delays up to 4000 ms and a BER ≤ 10^−6^. When a higher BER and/or a longer link delay are involved during transmission, LTP UDP SEC has the lowest goodput. UDP LTP PRIM shows a consistent goodput performance of around 1500 bytes/s at all the experimented link delays and channel error rates, implying that its performance is not affected by link delay and channel noise. In comparison, LTP UDP PRIM and LTP TCP SEC show obvious performance degradation with the increase in link delays at all three error rates.

The performance differences of the protocols are mainly caused by their design and operation differences, which are also investigated.

## Figures and Tables

**Table 1:** Experimental configuration.

Experiment Factors	Settings
Protocol implementation	Interplanetary Overlay Network (ION) v4.1.1 by Jet Propulsion Laboratory (JPL) [57]
Protocol configurations	LTP LTP PRIM UDP LTP PRIM LTP UDP SEC LTP TCP SEC (with SACK option enabled for TCP) (See Figure 3 for the details)
LTP segment settings	100% red data
LTP segment size	1000 bytes
MTU size	1500 bytes
Nominal Block Size (NBS) × Maximum number of sessions (NOS)	1800 (bytes) × 32 (Selected based on tuning the protocol for the maximum goodput)
Maximum and default sender and receiver buffer size	16 Mbytes for TX, MX and RX
Operating system	Fedora Linux 38 (kernel 6.2)
BER	0, 10^−6^, and 10^−5^ (to emulate different channel qualities)
One-way link latency	Nine different delays selected in a range of 1280~5000 ms (Typical cislunar link delays chosen according to [24])
Channel rate on each link	115,200 bit/s
Link disruption	Not introduced
File size for simulation	1 Mbyte
Sample size	16 repetitive runs

**Table 2:** Known data rates and link delay in actual lunar missions.

Mission Name	Data Link Bit Rate		One-Way Link Delay
	Data Link	Bit Rate	
Apollo 11~17	To Earth	51.2 kbps (S-band frequency) [58] (pp. 9–10)	~1.7 s
Lunar Reconnaissance Orbiter (LRO)	To Earth	100 kbps~100 Mbps (S- and K-band) [59]	~1.7 s

**Table 3:** List of graphical notations.

Notations	Descriptions
*P*-LTP TCP SEC vs. LTP UDP SEC	*p*-value for goodput difference between LTP TCP SEC and LTP UDP SEC
*P*-LTP TCP SEC vs. UDP LTP PRIM	*p*-value for goodput difference between LTP TCP SEC and UDP LTP PRIM
*P*-LTP UDP SEC vs. UDP LTP PRIM	*p*-value for goodput difference between LTP UDP SEC and UDP LTP PRIM
*P*-Threshold	Threshold *p*-value (set to 0.05) for a comparison reference
LTP TCP SEC—LTP UDP SEC	Goodput difference between LTP TCP SEC and LTP UDP SEC
LTP TCP SEC—UDP LTP PRIM	Goodput difference between LTP TCP SEC and UDP LTP PRIM
LTP UDP SEC—UDP LTP PRIM	Goodput difference between LTP UDP SEC and UDP LTP PRIM

**Table 4:** Rough performance ranking of investigated DTN protocols.

Performance Ranking	BER = 0	BER = 10^−6^	BER = 10^−5^
1	LTP LTP PRIM	LTP LTP PRIM	LTP LTP PRIM
2	LTP TCP SEC	LTP TCP SEC	LTP TCP SEC (Delay < 2500 ms) UDP LTP PRIM (Delay ≥ 2500 ms)
3	LTP UDP SEC (Delay < 3500 ms) UDP LTP PRIM (Delay > 4000 ms)	LTP UDP SEC (Delay < 3500 ms) UDP LTP PRIM (Delay > 3500 ms)	UDP LTP PRIM (Delay < 2500 ms) LTP TCP SEC (Delay ≥ 2500 ms)
4	UDP LTP PRIM (Delay < 3500 ms) LTP UDP SEC (Delay ≥ 3500 ms)	UDP LTP PRIM (Delay ≤ 3500 ms) LTP UDP SEC (Delay > 3500 ms)	LTP UDP SEC

## Data Availability

Dataset available on request from the authors.
